# Diet composition and resource overlap of sympatric native and introduced salmonids across neighboring streams during a peak discharge event

**DOI:** 10.1371/journal.pone.0280833

**Published:** 2023-01-24

**Authors:** Tanner L. Cox, Michael J. Lance, Lindsey K. Albertson, Michelle A. Briggs, Adeline J. Dutton, Alexander V. Zale

**Affiliations:** 1 Montana Cooperative Fishery Research Unit, Montana State University, Bozeman, MT, United States of America; 2 Department of Ecology, Montana State University, Bozeman, MT, United States of America; 3 U.S. Geological Survey, Montana Cooperative Fishery Research Unit, Montana State University, Bozeman, MT, United States of America; Complutense University of Madrid: Universidad Complutense de Madrid, SPAIN

## Abstract

Species assemblages composed of non-native and native fishes are found in freshwater systems throughout the world, and interactions such as interspecific competition that may negatively affect native species are expected when non-native species are present. In the Smith River watershed, Montana, rainbow trout were introduced by 1930. Native mountain whitefish and non-native rainbow trout have presumably occurred in sympatry since the introduction of rainbow trout; however, knowledge about how these two species compete with one another for food resources is sparse. We quantified diet compositions of rainbow trout and mountain whitefish in the mainstem Smith River and in a tributary to the Smith River—Sheep Creek—to determine the degree of overlap in the diets of mountain whitefish and rainbow trout in the Smith River and between the mainstem Smith River and a tributary stream. Rainbow trout and mountain whitefish had generalist feeding strategies, which probably contribute to the amicable coexistence of these species. Diet overlap between rainbow trout and mountain whitefish was high (Pianka’s index value = 0.85) in the Smith River and moderate in Sheep Creek (Pianka’s index value = 0.57). Despite overlap in diets, some resource partitioning may alleviate resource competition (e.g., rainbow trout consumed far more Oligochaeta than mountain whitefish but fewer Brachycentridae and Chironomidae). Diet composition of rainbow trout and mountain whitefish did not differ greatly between the Smith River and Sheep Creek. Prey categories most commonly used by mountain whitefish at the population and individual levels (i.e., Ephemeroptera and Trichoptera) are sensitive taxa and many species within these orders have experienced extinctions and population declines. Therefore, future changes in resource availability or competition could be of concern.

## Introduction

Non-native fishes have been introduced to freshwater systems throughout the globe, often with dramatic consequences for the receiving ecosystems [1]. Non-native fishes can harm native fish populations through predation, competition, and hybridization [2, 3]. Throughout the western United States, the introductions of non-native salmonids such as rainbow trout (*Oncorhynchus mykiss*), brook trout (*Salvelinus fontinalis*), and brown trout (*Salmo trutta*) have displaced or reduced the abundances of native salmonids [4–6]. Rainbow trout have been widely introduced throughout the world because they are a preferred sport fish that can be successfully cultured and acclimate to a wide array of conditions (e.g., lentic and lotic systems). Rainbow trout have been particularly successful in habitats outside of their native range and often become dominant after outcompeting native salmonid species [7]. Understanding how native and introduced salmonids compete for resources such as food and habitat is important for ensuring the persistence of native fishes.

Investigating the feeding ecology of fishes is fundamental for understanding species interactions, population dynamics, trophic structure, and energy transfer both within and between ecosystems [8]. Comparing the feeding ecology of species can shed light on the ways in which two species may compete for or share available food resources. For example, diet overlap can indicate resource sharing; however, the same prey types may be consumed in different portions of the water column or at different times [9]. Quantifying and characterizing fish diets are the first steps in evaluating resource sharing and provide managers with vital information about habitat requirements with implications for species conservation and restoration. Investigating feeding ecology can inform our understanding of how introduced and native salmonids interact within a watershed.

Interactions between native and introduced fishes may vary among locations within a watershed. Tributaries are important components of watersheds and are often subject to fewer anthropogenic influences, such as flow regulation, than mainstem habitats [10]. Tributaries increase habitat heterogeneity, provide spawning and rearing habitat, serve as thermal refugia during warm summer months, and often support abundant food resources for fishes [11–13]. Communities can have stark differences in the abundance and diversity of macroinvertebrate species between a mainstem stream and tributaries of it [14], which may induce changes in patterns of competition between fish species between mainstem and tributary habitats. Therefore, characterizing diet overlap in both mainstem and tributaries—particularly during migrations—may provide additional insight about interactions between competing species.

Mountain whitefish (*Prosopium williamsoni*) are a salmonid native throughout the northern Rocky Mountains. They persist in much of their native range and often make up a large portion of fish biomass and abundance [15, 16]. Because they are often not considered a desirable game species, mountain whitefish remain relatively understudied, with little information on population trends, diet, and habitat requirements (but see [17, 18]). Although extensive literature exists on the effects of introduced salmonids on native cutthroat trout and bull trout [19–21], studies identifying interactions between mountain whitefish and introduced salmonids, such as rainbow trout, are relatively rare. Understanding the interactions between mountain whitefish and introduced species may provide insights into population dynamics, such as why mountain whitefish remain abundant in certain locations where non-native salmonids are also abundant. In addition, knowledge of species interaction could help managers conserve mountain whitefish populations as introduced species continue to spread throughout the native range of mountain whitefish.

We investigated the feeding ecology of an introduced salmonid, the rainbow trout, and an understudied native salmonid, the mountain whitefish, in the Smith River watershed, a large, unfragmented watershed located in central Montana. Mountain whitefish have been documented entering tributaries during the spring [22, 23]. We hypothesized that they may be moving into tributaries to access seasonally available food resources because these movements occur outside of their annual spawning period and during periods when water temperatures are not stressful. Therefore, during the spring of 2016, we sampled the diets of mountain whitefish and rainbow trout to understand the patterns of food items consumed by both species during the spring and compare diets between the species. Our specific objectives were (1) to determine the degree of overlap in the diets of mountain whitefish and rainbow trout in the Smith River watershed and (2) to determine if diets of these two species vary between the mainstem Smith River and a tributary stream. Our findings provide some of the first evidence of diet overlap and potential competition between native mountain whitefish and non-native rainbow trout.

## Materials and methods

### Study area

**Fig 1:**
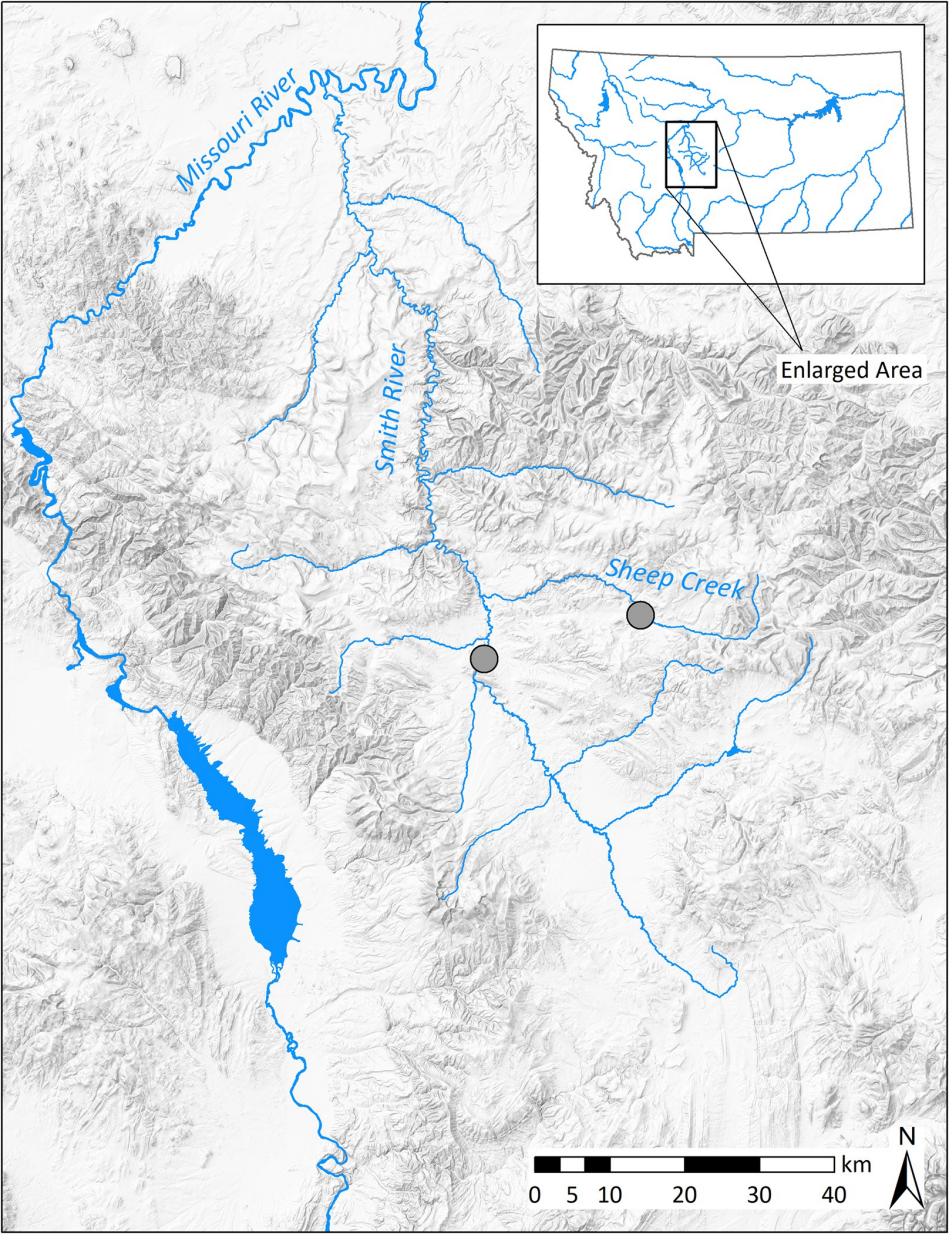
Study area map. The Smith River watershed in central Montana. Sampling reaches are denoted by grey circles. Graphic created using ESRI software (Redlands, CA) and open-access data from the Montana State Library including Montana Major Streams and Lakes (1993) and Grayscale Shaded Relief Image of Montana, 30-meter (2002).

This study took place in two streams in central Montana, USA. The Smith River originates near White Sulphur Springs, Montana, and flows 195 km northwest to its confluence with the Missouri River near Great Falls, Montana (Fig 1). At its headwaters, the Smith River flows through a broad, agricultural valley. The river then flows through a canyon in the Little Belt Mountains, followed by a prairie region as it approaches its confluence with the Missouri River [24]. Sheep Creek is one of the primary tributaries of the Smith River. Sheep Creek flows 59 km west from its headwaters in the Little Belt Mountains to its confluence with the Smith River, at the upstream portion of the canyon region. Both streams follow a typical hydrograph for snowmelt runoff streams, with peak discharge occurring in late spring to early summer and discharge gradually declining to base flows in mid-summer.

Native salmonids in the Smith River watershed include mountain whitefish and westslope cutthroat trout (*Oncorhynchus clarkii lewisi*; mainly isolated in small tributaries). Other native fishes include white sucker (*Catostomus commersonii*), longnose sucker (*C*. *catostomus*), mountain sucker (*C*. *platyrhynchus*), burbot (*Lota lota*), stonecat (*Noturus flavus*), Rocky Mountain sculpin (*Cottus bondi*), and longnose dace (*Rhinichthys cataractae*). Three non-native fishes were intentionally introduced in the twentieth century for recreational angling: brown trout, rainbow trout, and brook trout. The most abundant fishes in the watershed are mountain whitefish, rainbow trout, and brown trout [25, 26].

### Data collection

Diet sampling was conducted on May 24–25, 2016, during spring runoff. Sampling was performed under the auspices of Montana State University institutional animal care and use protocol 2014–50 and scientific collectors permit 12–2016 issued by the Montana Department of Fish, Wildlife and Parks to Alexander Zale. We sampled one reach (about 2 km long) in each stream. The sampling reach on the Smith River was in the headwater portion of the watershed, upstream of the confluence with Sheep Creek. The sampling reach on Sheep Creek was located about 26 km upstream of the confluence of Sheep Creek and the Smith River. To capture fish, we used raft electrofishing in the Smith River and raft or barge electrofishing in Sheep Creek. A single pass was conducted along each site. We used a Coffelt VVP electrofisher and a smooth DC waveform. Output voltage varied between the sites; it was adjusted to the minimum voltage necessary to achieve good capture efficiency. Output amperage was maintained at five amperes or less. In Sheep Creek, a mobile anode configuration was used, and in the Smith River, the anodes were fixed booms attached to the electrofishing raft. The mobile-anode and boom-anode configurations were both maneuvered to effectively cover the width of the stream being fished. Fish were anaesthetized using Aqui-S 20E (Aqui-S New Zealand Ltd.; 10 to 20 mg/L titrated to efficacy) prior to handling and were allowed to recover in fresh water prior to release. We measured the total length (TL) of each fish and collected diets using a standard gastric-lavage technique with a modified garden sprayer with an attached 6.35-mm polypropylene tube for insertion into the esophagus of the fish. Stomach contents were collected into a tub, rinsed into a 500-μm mesh sieve, and preserved in 95% ethanol. Diets were collected from 11 mountain whitefish (TL range = 306–396 mm) and six rainbow trout (TL range = 285–415 mm) from the Smith River and 20 mountain whitefish (TL range = 244–408) and 20 rainbow trout (TL range = 138–395 mm) from Sheep Creek. In the laboratory, aquatic insects were identified to family, terrestrial insects were grouped and not identified further, and other diet items were identified to order using standard methods at 10×–100× magnification under a dissecting microscope [27]. We quantified the number of individuals and the proportion by count of each diet category in every fish diet. We evaluated diets with diet items categorized at both the family and order levels to understand hierarchical patterns in diet composition.

### Data analysis

We used generalized linear models (GLM) with a quasi-binomial distribution and a logit-link function to model the proportion of each of eight prey types in the diets of mountain whitefish and rainbow trout. A quasi-binomial distribution is appropriate for proportional data with overdispersion. Our response variable was the proportion of items from the modelled diet category in the diet, weighted for the total number of items in the diet. Our explanatory variables were species, stream, the interaction between species and stream, and fish length. We constructed nine models for each response variable, ranging from a global model containing all explanatory variables to a null model containing only an intercept term. We used QAICc, which incorporates a dispersion parameter, to select a top model for each prey type [28]. From all model options, we selected the most parsimonious model with ΔQAICc ≤ 2 as the final model for each prey type (S1 Table). We then used model output to estimate the predicted probability of consumption of each prey type by mountain whitefish and rainbow trout in the Smith River and Sheep Creek. For models that included length, we used the median fish length in our dataset to calculate probability estimates (306 mm). All data analysis occurred in Program R version 4.1.0.

We used Pianka’s index of niche overlap to quantify diet overlap between mountain whitefish and rainbow trout in the Smith River and Sheep Creek. Niche overlap *O*_*jk*_ is defined as $O_{jk}=O_{kj}=\frac{\sum_{i}^{n}p_{ij}p_{ik}}{\sqrt{\sum_{i}^{n}{p_{ij}}^{2}\sum_{i}^{n}{p_{ik}}^{2}}}$ where *p*_*ij*_ and *p*_*ik*_ are the proportions of the *i*^*th*^ resource used by the *j*^*th*^ and *k*^*th*^ species, respectively [29]. Proportions were calculated using quantities of diet items within each category. Values of *O*_*jk*_ range from 0 to 1, with 0 indicating no diet overlap and 1 indicating complete diet overlap. Values of *O*_*jk*_ ≥ 0.60 are commonly used to indicate high diet overlap (e.g., see Novakowski; 2008 [30]).

To investigate patterns in feeding ecology, we calculated both frequency of occurrence (*F*_*i*_) and prey-specific abundance (*P*_*i*_) for both fish species in the Smith River and Sheep Creek. Frequency of occurrence is defined as $F_{i}=(\frac{N_{i}}{N})$, and *P*_*i*_ is defined as $P_{i}=(\frac{\sum S_{i}}{\sum S_{t_{i}}})$ where *N*_*i*_ is the number of individuals with prey *i* in their stomach and *N* is the number of individuals with stomach contents, *S*_*i*_ is the number of diet items made up of prey *i*, and $S_{t_{i}}$ is the total stomach content (i.e., total quantity of diet items) in only those individuals with prey *i* in their stomach. Presenting these results in a bivariate plot provides information about feeding strategies, prey importance, and variability in diets [31]. Prey categories that have a high prey-specific abundance indicate that individuals specialize on the prey type, whereas low prey-specific abundance results from more opportunistic, generalist feeding strategies. Prey categories that have high frequency of occurrence indicate that most individuals in the population consume the prey type, whereas low frequency of occurrence indicates that few individuals make use of the prey type.

## Results

**Fig 2:**
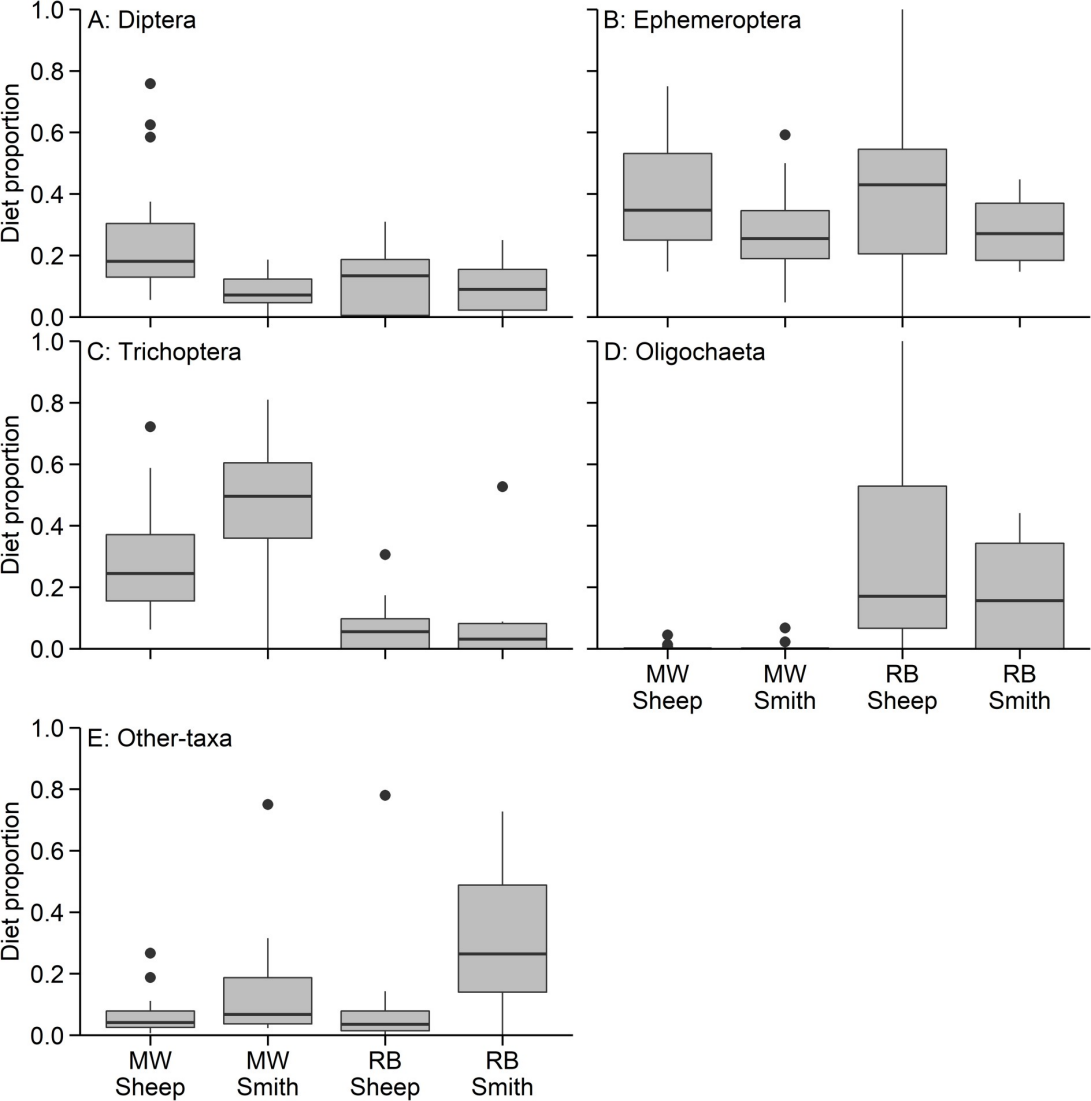
Diet categories grouped by order. Standard boxplots showing proportions of diet item from the specified category found in mountain whitefish (MW) and rainbow trout (RB) in Sheep Creek and the Smith River. Panels show proportions of (A) Diptera; (B) Ephemeroptera; (C) Trichoptera; (D) Oligochaeta; and (E) any other taxa pooled. Box ends represent the 25^th^ and 75^th^ quantiles, horizontal lines are the medians, the upper whisker extends to the largest observation no further than 1.5 × interquartile range (IQR) from the 75^th^ quantile, and the lower whisker extends to the smallest observation no further than 1.5 × IQR from the 25^th^ quantile.

**Fig 3:**
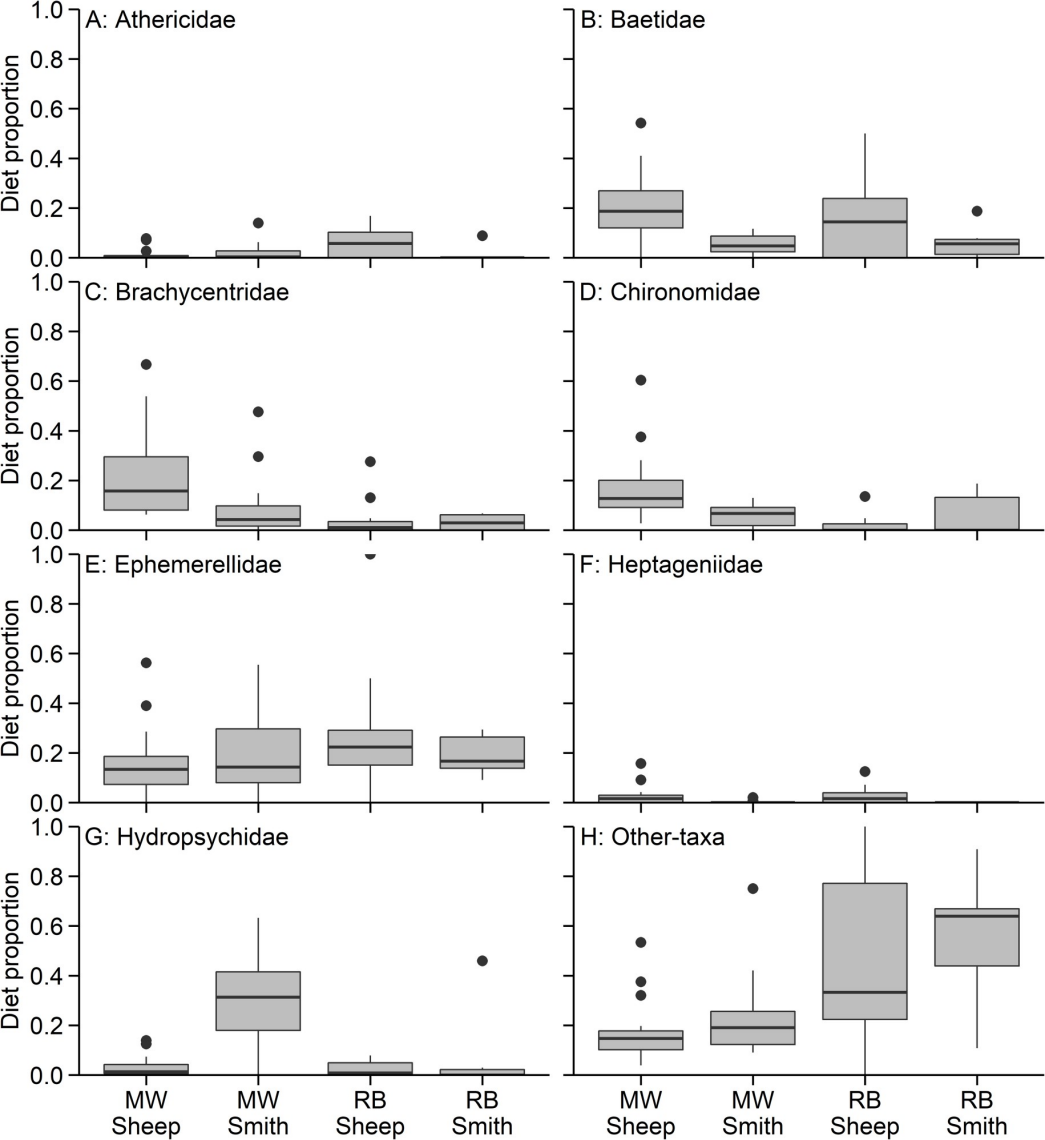
Diet categories grouped by family. Boxplots showing proportions of diet items from the specified category (family) found in mountain whitefish (MW) and rainbow trout (RB) diets in Sheep Creek and the Smith River. Panels show proportions of (A) Athericidae; (B) Baetidae; (C) Brachycentridae; (D) Chironomidae; (E) Ephemerellidae; (F) Heptageniidae; (G) Hydropsychidae; and (H) all other taxa pooled. Box ends represent 25^th^ and 75^th^ quantiles, horizontal lines are the medians, the upper whisker extends to the largest observation no further than 1.5 × interquartile range (IQR) from 75^th^ quantile, and the lower whisker extends to the smallest observation no further than 1.5 × IQR from 25^th^ quantile.

Rainbow trout and mountain whitefish in the Smith River and Sheep Creek (i.e., across both species and rivers) consumed a wide array of prey types, but mayflies (Ephemeroptera), caddisflies (Trichoptera), segmented worms (Oligochaeta), and midges (Diptera) were found in most diets (Fig 2). The proportion of diets composed of invertebrates from different families varied between species and locations where fish were captured. In the Smith River, mountain whitefish consumed primarily Hydropsychidae, Ephemerellidae, and Brachycentridae; these taxa made up an average of 30.2%, 20.7%, and 10.2% of total diet items, respectively (Fig 3). In Sheep Creek, mountain whitefish diets were generally composed of Brachycentridae (22.4%), Baetidae (21.1%), Chironomidae (16.8%), and Ephemerellidae (16.3%). Diets of rainbow trout in the Smith River were composed of 19.1% Ephemerellidae, 18.4% Oligochaeta, and 11.8% fish eggs, and in Sheep Creek, rainbow trout most commonly consumed Oligochaeta (30.2%), Ephemerellidae (25.0%), and Baetidae (14.8%).

The occurrence of diet items from some categories did not vary between the two species but did vary between the two sites. Baetidae and Heptageniidae made up a higher proportion of fish diets in Sheep Creek than in the Smith River (Tables 1 and 2). However, Heptageniidae made up a small portion of fish diets in both streams. Modeling results indicate the probability of a fish consuming Baetidae in Sheep Creek was 0.20 (95% CI 0.17–0.24), compared to 0.04 in the Smith River (95% CI 0.02–0.08). Both Hydropsychidae and Ephemerellidae constituted higher proportions of fish diets in the Smith River than in Sheep Creek (Tables 1 and 2). Hydropsychidae were commonly consumed in the Smith River, where they made up a quarter of the total diet items (95% CI 0.19–0.31) but were rarely consumed in Sheep Creek (proportion = 0.03, 95% CI 0.02–0.05). Ephemerellidae, which were an important diet category in both streams, were more commonly consumed in the Smith River (0.26 of total diet items; 95% CI 0.20–0.33) than in Sheep Creek (0.16 of total diet items; 95% CI 0.13–0.20).

**Table 1:** Models of eight most consumed diet categories. Summary of model results for the eight most commonly consumed diet categories. Dashes indicate parameters that were not included in the final model. Coefficient estimates, in the first column under each explanatory variable, are reported on the log-odds scale. Baseline categories were Sheep Creek for the stream variable and mountain whitefish for the species variable. Adjusted deviance explained for each model was Athericidae = 0.40, Baetidae = 0.40, Brachycentridae = 0.32, Chironomidae = 0.52, Ephemerellidae = 0.08, Heptageniidae = 0.19, Hydropsychidae = 0.57, and Oligochaeta = 0.56.

Diet category (response variable)	Intercept			Stream			Species			Length			Stream × Species		
		t-value	p-value		t-value	p-value		t-value	p-value		t-value	p-value		t-value	p-value
Athericidae	-4.965	-10.39	<0.001	1.354	2.33	0.024	2.474	4.77	<0.001	—	—	—	-3.053	-2.47	0.017
Baetidae	-1.356	-12.88	<0.001	-1.780	-5.18	<0.001	—	—	—	—	—	—	—	—	—
Brachycentridae	-1.095	-6.55	<0.001	-0.855	-2.58	0.013	-1.815	-3.99	<0.001	—	—	—	—	—	—
Chironomidae	1.642	1.74	0.087	-0.346	-1.17	0.246	-2.860	-6.09	<0.001	-0.010	-3.15	0.002	2.645	3.47	0.001
Ephemerellidae	-1.635	-11.41	<0.001	0.584	2.47	0.016	—	—	—	—	—	—	—	—	—
Heptageniidae	-3.614	-18.19	<0.001	-2.254	-2.39	0.020	—	—	—	—	—	—	—	—	—
Hydropsychidae	-3.535	-13.40	<0.001	2.413	7.86	<0.001	—	—	—	—	—	—	—	—	—
Oligochaeta	-9.441	-4.48	<0.001	-2.118	-2.19	0.033	4.267	4.90	<0.001	0.015	2.53	0.014	—	—	—

**Table 2:** Estimated food item proportions. The estimated proportions of total food items in the diets of mountain whitefish and rainbow trout in the Smith River and Sheep Creek by taxon. Estimates are derived from generalized linear models, and confidence intervals (95%) are shown in parentheses. Only the eight most common taxa in fish diets are listed.

Diet category	Mountain whitefish Smith River	Mountain whitefish Sheep Creek	Rainbow trout Smith River	Rainbow trout Sheep Creek
Athericidae	0.026	0.007	0.015	0.077
	(0.014–0.049)	(0.003–0.018)	(0.002–0.111)	(0.053–0.110)
Baetidae	0.042	0.205	0.042	0.205
	(0.022–0.077)	(0.173–0.241)	(0.022–0.077)	(0.173–0.241)
Brachycentridae	0.125	0.251	0.023	0.052
	(0.075–0.200)	(0.195–0.317)	(0.008–0.061)	(0.023–0.111)
Chironomidae	0.162	0.214	0.135	0.015
	(0.099–0.253)	(0.177–0.258)	(0.047–0.332)	(0.007–0.036)
Ephemerellidae	0.259	0.163	0.259	0.163
	(0.196–0.334)	(0.129–0.205)	(0.196–0.334)	(0.129–0.205)
Heptageniidae	0.003	0.026	0.003	0.026
	(0.0004–0.018)	(0.018–0.039)	(0.0004–0.018)	(0.018–0.039)
Hydropsychidae	0.246	0.028	0.246	0.028
	(0.192–0.308)	(0.017–0.047)	(0.192–0.308)	(0.017–0.047)
Oligochaeta	0.001	0.007	0.056	0.332
	(0.00007–0.010)	(0.002–0.031)	(0.011–0.236)	(0.213–0.477)

Patterns of consumption of some diet categories varied between both sites and between both species. The proportion of Brachycentridae in fish diets was higher in mountain whitefish and in Sheep Creek (Tables 1 and 2) and Brachycentridae composed small portions of rainbow trout diets in both streams. The proportion of fish diets composed of Oligochaeta differed by species, stream, and fish length. Oligochaeta were larger portions of diets of rainbow trout, fish in the Sheep Creek, and larger fish (Tables 1 and 2). The differences in composition of diets were greater between species than between streams. The predicted proportion of rainbow trout diets composed of Oligochaeta was 0.06 (95% CI 0.01–0.24) in the Smith River and 0.33 (95% CI 0.21–0.48) in Sheep Creek, whereas the predicted proportion of mountain whitefish diets composed of Oligochaete worms was < 0.01 in both streams. The model for the proportion of Chironomidae in fish diets included length and the interaction between species and stream (Tables 1 and 2). The estimated proportion of Chironomidae in fish diets was highest for mountain whitefish in the Sheep Creek at 0.21 (95% CI 0.18–0.26) followed by mountain whitefish in Smith River, rainbow trout in Smith River, and rainbow trout in the Sheep Creek (Table 2). Additionally, smaller fish had a higher proportion of Chironomidae in their diets. The estimated proportion of Athericidae in fish diets was generally low (Table 1), and the proportion of Athericidae in diets varied by stream and species with an interaction between the two predictors (Table 2).

In the Smith River, diet overlap was high between mountain whitefish and rainbow trout (Pianka’s index value = 0.85), but diet overlap between the two species was lower in Sheep Creek (Pianka’s index value = 0.57, Table 3). Whereas a Pianka’s index value of 0.57 does not indicate high overlap, this value indicates some overlap in diets between mountain whitefish and rainbow trout in Sheep Creek. Rainbow trout had high diet overlap between Sheep Creek and the Smith River, whereas mountain whitefish had lower overlap between the two streams.

**Table 3:** Niche overlap. Pianka’s index of niche overlap comparing diets of mountain whitefish and rainbow trout in Sheep Creek and the Smith River. Values ≥ 0.60 indicate high diet overlap.

	Mountain whitefish Sheep Creek	Mountain whitefish Smith River	Rainbow trout Sheep Creek	Rainbow trout Smith River
Mountain whitefish Sheep Creek	—	0.59	0.57	—
Mountain whitefish Smith River	0.59	—	—	0.85
Rainbow trout Sheep Creek	0.57	—	—	0.72
Rainbow trout Smith River	—	0.85	0.72	—

**Fig 4:**
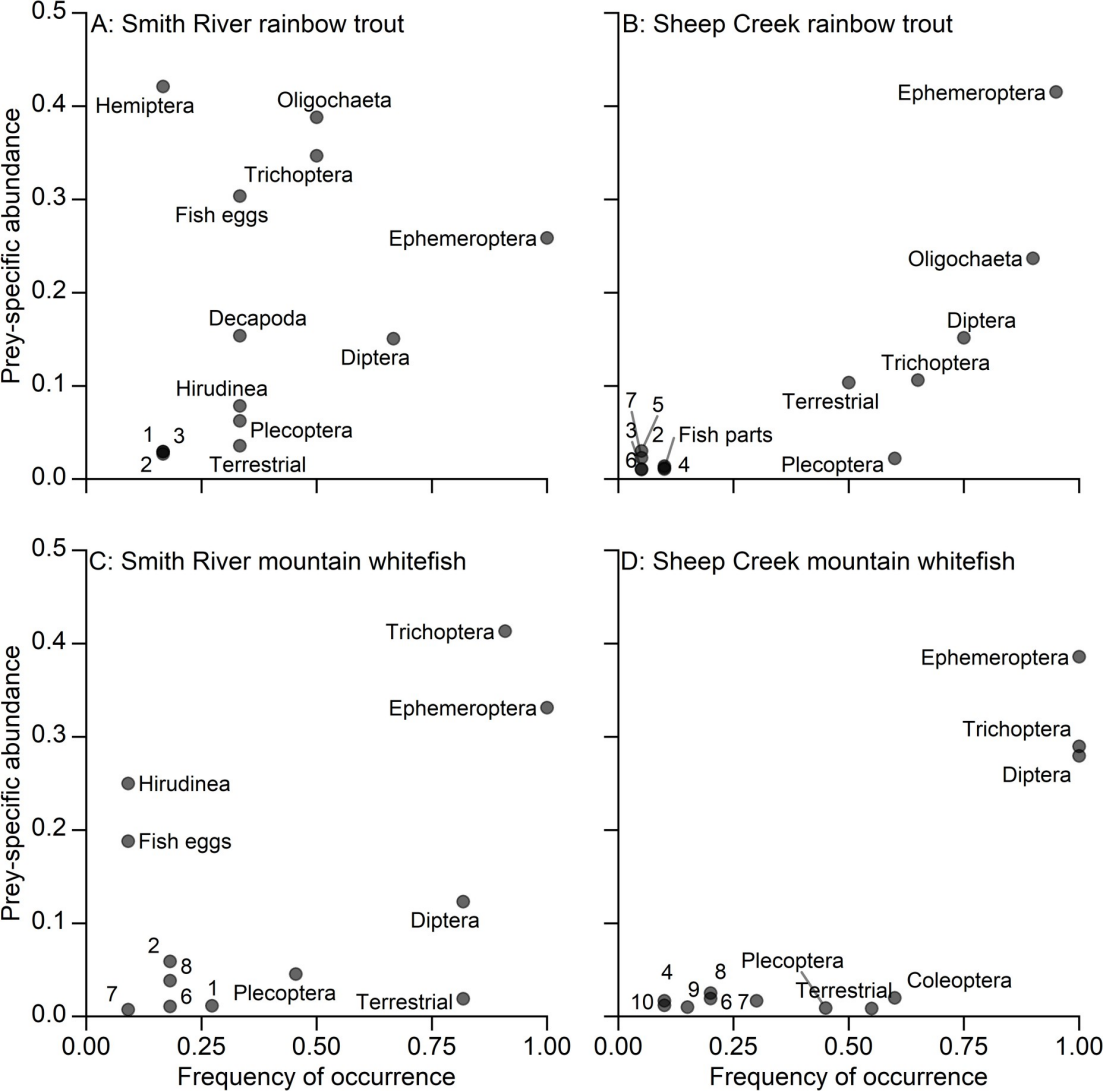
**Feeding strategy diagram.** Feeding strategy diagram of the modified Costello method according to Amundsen et al. (1996) [31]. Prey-specific abundances and frequencies of occurrence in the diets of mountain whitefish (MW) and rainbow trout (RB) in the Smith River and Sheep Creek. Numbered diet categories are 1: Fish larvae; 2: Coleoptera; 3: Amphipoda; 4: Scorpaeniformes; 5: Hemiptera; 6: Gastropoda; 7: Diptera pupae; 8: Oligochaeta; 9: Acari; 10: Neuroptera. Panels show (A) rainbow trout in the Smith River; (B) rainbow trout in Sheep Creek; (C) mountain whitefish in the Smith River; and (D) mountain whitefish in Sheep Creek.

**Fig 5:**
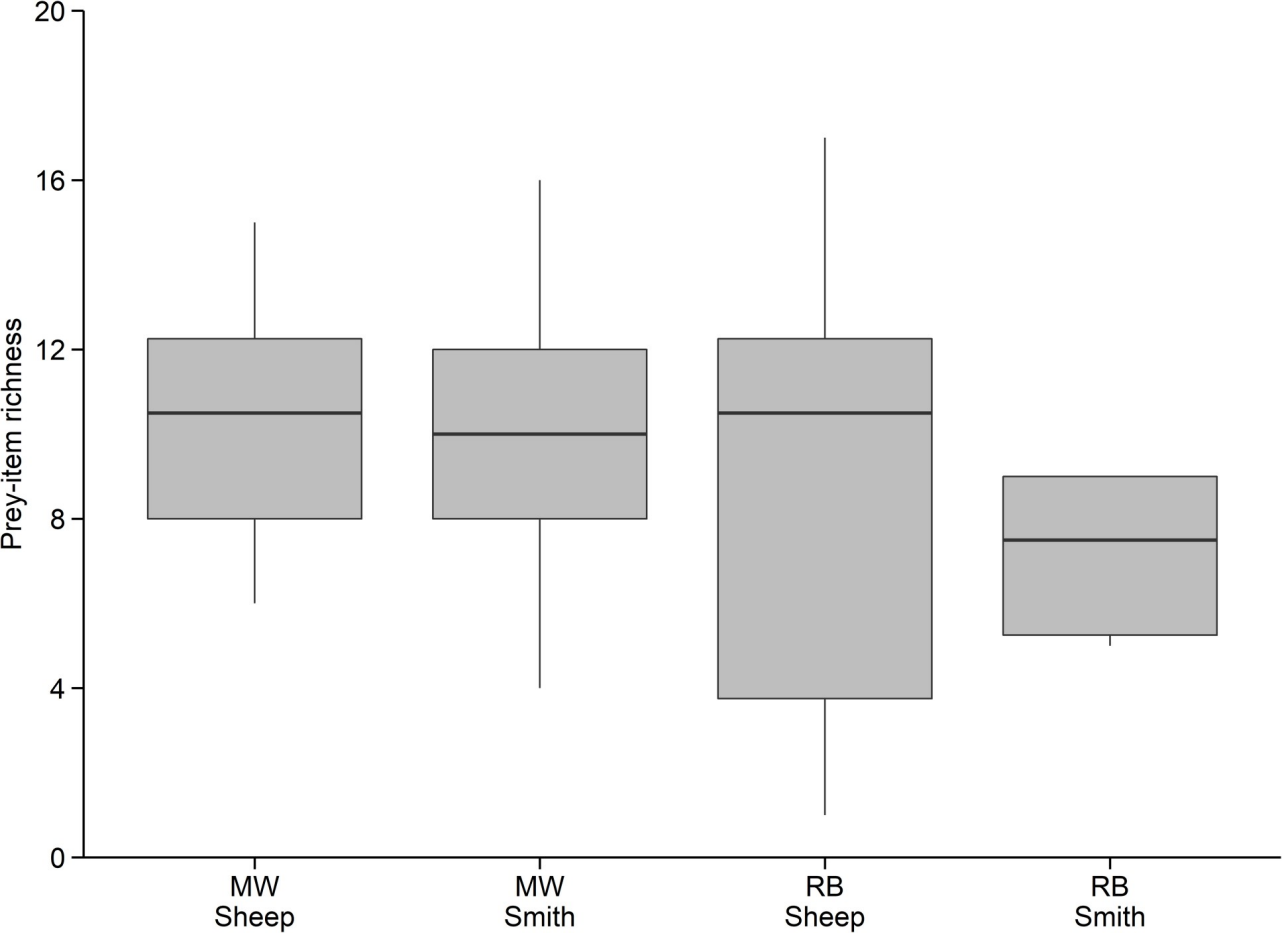
Prey-item richness. Prey-item richness (i.e., number of unique prey-items) in the diets of mountain whitefish (MW) and rainbow trout (RB) in the Smith River and Sheep Creek. Box ends represent 25^th^ and 75^th^ quantiles, horizontal lines are the medians, the upper whisker extends to the largest observation no further than 1.5 × interquartile range (IQR) from 75^th^ quantile, and the lower whisker extends to the smallest observation no further than 1.5 × IQR from 25^th^ quantile.

Mountain whitefish and rainbow trout in the Smith River and Sheep Creek exhibited generalist feeding strategies with prey-specific abundance of prey items remaining below 0.50 in all cases (Fig 4). However, there were dominant prey types on the population level. For some prey types, frequency of occurrence was high (up to 1.0) and prey-specific abundance was low (< 0.5), indicating these prey types were consumed by most individuals but made up a small proportion of the diet of those individuals. Many prey types had low frequency of occurrence (< 0.5) indicating they were not widely consumed on a population level. Furthermore, diets of all groups of fish included infrequently consumed taxa on the population and individual levels. Rainbow trout in the Smith River had a slightly more specialized feeding strategy than the other groups of fish sampled. Hemiptera (true bugs) and Oligochaeta had a higher prey-specific abundance and a lower frequency of occurrence (Fig 4; panel A), indicating that these diet categories made up a large portion of the diet of relatively few individuals in the population. Individual fish consumed up to 17 different prey items (range 1–17), and the median prey-item richness was eight or more for all groups of fish (Fig 5). Among all fishes, the median prey-item richness was 10.

## Discussion

During spring runoff, mountain whitefish and rainbow trout in the Smith River and Sheep Creek fed on a variety of taxa and showed moderate to high overlap in diets interspecifically as well as conspecifically between locations within the watershed. Mountain whitefish most frequently consumed aquatic invertebrates, particularly mayflies, caddisflies, and midges as seen in previous studies, although mountain whitefish in other rivers consumed fewer mayflies [32, 33] and higher quantities of midges and stoneflies (order Plecoptera) than in the Smith River watershed. Previous research indicated that mountain whitefish feed in both the drift and the benthos, and the taxa most frequently consumed by mountain whitefish in our study are found in both the drift and the benthos [32]. Mountain whitefish consumed small amounts of terrestrial insects, which is consistent with previous research showing that these fish only occasionally feed from the surface [33].

Although diet overlap between the two species was high in the Smith River and moderate in Sheep Creek during spring runoff, the generalist feeding strategies—including a large diversity of prey types consumed—exhibited by rainbow trout and mountain whitefish may reduce competition for food resources. Mountain whitefish tend to use similar food resources to other salmonids such as rainbow trout and cutthroat trout, resulting in niche overlap [33, 34]. However, similarity between diets of mountain whitefish and other salmonids does not explicitly indicate competition between species [34, 35]. Competition between species can be alleviated by abundant food resources and by subtle differences in feeding strategy that are not apparent from gut contents (e.g., although both species feed in the drift, mountain whitefish also feed in the benthos, and rainbow trout also feed at the surface). Therefore, different portions of the water column being used more commonly by each species may buffer overlap in diets like previously demonstrated in other species (e.g., in stream-dwelling Arctic charr [*Salvelinus alpinus*], Atlantic salmon [*Salmo salar*] and alpine bullhead [*Cottus poecilopus*]; [9]). Mountain whitefish in the Smith River watershed are adapted to coexist with other *Oncorhynchus* species since cutthroat trout are native to the Smith River watershed. Therefore, useful resource partitioning strategies and coping mechanisms that pre-date rainbow trout introductions may exist. Minor differences in diet composition by species indicated some partitioning of resources within the Smith River watershed. For example, rainbow trout consumed far more Oligochaeta than mountain whitefish but fewer Brachycentridae and Chironomidae. The ability of mountain whitefish to take advantage of numerous prey types may help the species remain abundant in watersheds where introduced salmonids have displaced other native salmonids, such as cutthroat trout. Nonetheless, fluctuations in availability of important diet categories along with other factors such as low flow and thermal limitations during late summer and early autumn are potential limiting factors that could be evaluated by future research.

Diets did not differ greatly between fish in the Smith River and Sheep Creek during spring runoff, and rainbow trout showed high overlap between the two locations. Although, stream was considered a useful predictor variable of probability of consumption for all prey types that were modeled. Baetidae were consumed more frequently in Sheep Creek, while Ephemerellidae and Hydropsychidae were consumed more frequently in the Smith River. These differences probably parallel differences in macroinvertebrate assemblages. Rainbow trout spawn in tributary habitats in the spring (i.e., when we were sampling), and spawning rainbow trout may have used the food resources available in Sheep Creek during their migration. Some mountain whitefish move into tributary streams in the spring [23, 24, 36], and this movement may be driven in part by a search for food resources. However, given the availability of diet resources in the mainstem Smith River and the generalist feeding strategy of mountain whitefish, a search for food resources may not be driving the movement of mountain whitefish into tributaries in this system. Interestingly, mountain whitefish in Sheep Creek did not feed on fish eggs or embryos despite a common conception that mountain whitefish enter tributaries in the spring specifically to feed on rainbow trout eggs or embryos and historical beliefs that interspecific competition and consumption of sportfish eggs indicated a need for reducing mountain whitefish density [37]. Movements into tributaries in the spring are probably influenced by factors beyond food resources, such as seeking refuge from high mainstem discharges and water velocities during runoff or impending warm mainstem water temperatures in summer [36].

The timing of our sampling, changes in prey availability, and relatively low sample sizes have implications for our results. Fish diet composition and diet overlap between fish species can change seasonally as the availability of various diet categories, such as terrestrial insects, changes [38, 39]. Food resources for fish may be relatively abundant during the spring, when water temperatures are increasing and flows dislodge benthic invertebrates into the drift, but many insect taxa have not yet emerged [40]. Food availability may be limited during the autumn and winter and thereby could alter feeding strategies and increase diet overlap and competition between mountain whitefish and rainbow trout [35]. Furthermore, variation in food types available throughout the year resulting from cyclical or episodic events (e.g., eggs from fish spawning events or hatching of macroinvertebrates) could alleviate or exacerbate competition between rainbow trout and mountain whitefish. We cannot fully address feeding strategies without quantification of prey availability or sampling events stratified by season. Therefore, additional research could more fully characterize the feeding ecology of these two species, feeding strategies of the species, and how competition between the species might vary throughout the course of a year. Low sample sizes—particularly for Smith River rainbow trout (n = 6) and Smith River mountain whitefish (n = 11)—may influence our results, and future research may be useful to validate our findings that include Smith River fishes. Replicating the analysis with prey biomass would allow the results to be interpreted in a bioenergetic-based framework.

Quantifying fish diets can help us predict how these species may respond to increasing anthropogenic effects and can inform conservation efforts for native fishes. Concerning declines in insect biomass and diversity have been detected around the globe including in protected areas [41, 42], and among aquatic insects, sensitive taxa such as Trichoptera and Ephemeroptera are especially imperiled [42]. These imperiled macroinvertebrate orders made up most of the diets of mountain whitefish and rainbow trout in the Smith River watershed, and the continued presence of robust insect populations is vital to the diversity of fish species and the persistence of these fishes in the watershed.

## Supporting information

S1 Table Model selection table. ΔQAICc for each of nine models for the eight most common diet categories in mountain whitefish and rainbow trout diets in the Smith River and Sheep Creek. We selected the most parsimonious model out of models with ΔQAICc ≤ 2, shown in boldface.
(PDF)

S1 File Data collected. Count data for each prey category by individual fish diet.
(CSV)
