# Supplementary material for: Diet composition and resource overlap of sympatric native and introduced salmonids across neighboring streams during a peak discharge event
Source: PLoS One. 2023 Jan 24;18(1):e0280833. doi: 10.1371/journal.pone.0280833 (PMC9873158; doi:10.1371/journal.pone.0280833)
Supplement: S1 Table — ΔQAICc for each of nine models for the eight most common diet categories in mountain whitefish and rainbow trout diets in the Smith River and Sheep Creek. We selected the most parsimonious model out of models with ΔQAICc ≤ 2, shown in boldface. (PDF) [file pone.0280833.s001.pdf]

Model parameters	Degrees of freedom	Athericidae	Baetidae	Brachycentridae	Chironomidae	Ephemerellidae	Heptageniidae	Hydropsychidae	Oligochaeta
Intercept only	1	28.64	37.63	23.69	55.66	3.91	8.42	72.77	45.77
Length	2	22.34	30.67	21.44	57.71	5.82	8.78	49.98	43.34
Stream	2	30.37	**0.00**	22.49	54.07	**0.00**	**0.00**	**0.00**	42.52
Length + stream	3	22.98	2.11	9.53	55.62	0.77	2.20	1.08	43.74
Species	2	5.31	39.86	6.36	20.35	5.90	9.72	69.68	3.07
Length + species	3	7.57	28.90	8.68	5.12	7.32	11.03	52.23	3.38
Stream + species	3	7.36	0.69	**1.19**	12.80	1.15	2.18	2.21	4.55
Length + stream + species	4	9.27	3.02	0.00	6.75	2.86	4.15	3.04	**0.00**
Stream × species	4	**0.00**	2.60	3.37	8.75	0.77	4.20	3.23	6.88
Length + stream × species	5	2.48	4.94	2.46	**0.00**	3.13	6.19	4.87	1.91
